# Re: Simplified Fournier's gangrene severe index score (SFGSI)

**DOI:** 10.1590/S1677-5538.IBJU.2017.0535

**Published:** 2018

**Authors:** Sora Yasri, Viroj Wiwanitkit

**Affiliations:** 1 KMT Primary Care Center, Bangkok Thailand;; 2 Hainan Medical University, China

*To the editor,*

We read the publication on “Risk factors for mortality in Fournier's gangrene in a general hospital: use of simplified Fournier's gangrene severe index score (SFGSI)” with great interest (1). Tenório et al. concluded that “The main advantage is easy applicability because it contains only three parameters and can be used immediately after patient's admission” (1). Indeed, as noted by Yeniyol et al., “Patients’ metabolic status and the extent of disease at presentation is an important factor in the prognosis of Fournier's gangrene” (2). There are some concerns on this report. First, the score cannot be immediately used; it has to wait for gathering the information if there is no pre-admission complete clinical history and laboratory data. Second, the mentioned variables are also affected by other possible concomitant disorders. For example, hemoglobin can be affected by any inherited and non-inherited anemic disorders.
